# Analysis and experiment of a positioning and pointing mechanism based on the stick–slip driving principle

**DOI:** 10.3389/fnbot.2025.1567291

**Published:** 2025-05-15

**Authors:** Yongqi Zhu, Juan Li, Jianbin Huang, Weida Li, Gai Liu, Lining Sun

**Affiliations:** ^1^Robotics and Micro-systems Center, School of Mechanical and Electrical Engineering, Soochow University, Suzhou, China; ^2^China Academy of Space Technology, Beijing, China

**Keywords:** positioning and pointing mechanism, piezoelectric ceramics, stick–slip driving, resolution, dynamic model

## Abstract

**Introduction:**

Traditional positioning and pointing mechanisms often face limitations in simultaneously achieving high speed and high resolution, and their travel range is typically constrained. To overcome these challenges, we propose a novel positioning and pointing mechanism driven by piezoelectric ceramics in this study. This mechanism is capable of achieving both high speed and high resolution by using two driving principles: resonance and stick–slip. This paper will focus on analyzing the stick–slip driving principle.

**Methods:**

We propose a configuration of the drive module within the positioning and pointing mechanism. By applying a low-frequency sawtooth wave excitation to the piezoelectric ceramics, the mechanism achieves high resolution based on the stick–slip driving principle. First, a simplified dynamic model of the drive module is established. The motion process of the drive module in stick–slip driving is divided into the stick phase and slip phase. With static and transient dynamic analyses conducted for each phase, the relationship between the output shaft angle, resolution, and driving voltage is derived. It is observed that during the stick phase, the output shaft angle and the driving voltage exhibit an approximately linear relationship, while in the slip phase, the output shaft angle and the driving voltage display nonlinearity due to impact forces and vibrations. Finally, a prototype of the positioning and pointing mechanism is designed, and an experimental platform is constructed to test the resolution of the prototype.

**Results:**

We construct a prototype of a dual-axis positioning and pointing mechanism composed of multiple drive modules and conduct resolution tests using two control methods: synchronous control and independent control. When synchronous control is used, the output shaft achieves a resolution of 0.38*μrad*, while with independent control, the resolution of the output shaft reaches 0.0276*μrad*.

**Discussion:**

The research results show that the positioning and pointing mechanism proposed in this study achieves high resolution through stick–slip driving principle, offering a novel approach for the advancement of such mechanisms.

## 1 Introduction

With the development of space rendezvous and docking, optical communication systems, high-precision observation systems, and other technologies, the demand for high-precision and fast-response tracking and pointing systems has become increasingly urgent. However, traditional positioning and pointing mechanisms often difficult to balance high resolution and high speed, necessitating improvements in their driving mechanisms.

Traditional positioning and pointing mechanisms can be categorized into multi-axis gimbal and fast steering mirrors (Wang et al., 2022; Wan et al., 2022; Tian et al., 2022). The main structure of a multi-axis gimbal consists of a mechanically rotating frame controlled by motors, which is suitable for applications requiring a large motion range but lower resolution. While multi-axis gimbal technology has been widely used, it still cannot achieve high pointing accuracy due to limitations in structure and driving methods. To enhance pointing accuracy, some researchers have proposed using a combination of multi-axis gimbals and precision adjustment mechanisms, where the precision adjustment mechanism typically employs piezoelectric or voice coil motor-driven FSM (Zhang et al., 2022; Hao et al., 2024; Kim, 2024) which have also garnered significant attention from the academic community. Kluk (2007) designed a FSM driven by a voice coil motor, with a rotation range of ±13*mrad* and an angular resolution of 0.2*μrad*. Fang et al. (2016) designed a piezoelectric-driven dual-axis FSM, with an inclination range of approximately 4*mrad*, a closed-loop bandwidth exceeding 120 *Hz*, a rotation range of ±2.5*mrad*, and a positioning accuracy of 13*μrad*. Each positioning and pointing technology has its own limitations and cannot simultaneously achieve key performance metrics such as high speed, high resolution, and large travel range.

Piezoelectric ceramics, as polycrystalline materials with piezoelectric effects, offer nanometer-level deformation precision, making them widely favored by researchers for precision positioning platforms in micro-operation and micro-positioning applications. These precision positioning platforms combine piezoelectric ceramics with compliant mechanisms, utilizing the elastic deformation of flexible hinges for guidance, enabling nanometer-scale positioning resolution (Liu et al., 2024; Zhong et al., 2019; Liu et al., 2024; Yin et al., 2023). Zhang et al. (2021) proposed a small-scale precision positioning platform driven by inertial stick–slip, with platform dimensions of 15 *mm* × 10 *mm* × 9.5 *mm*. Its linear velocity reaches 3.553 *mm*/*s*, and its angular velocity can attain 462.72*mrad*/*s*. Tang et al. (2021) designed a large-range nanometer positioning platform with a travel range of 1.035 *mm* × 1.035 *mm*, achieving an average tracking error within ±100 nm. Yin et al. (2023) developed a large-range, high-precision parallel positioning platform, which, when carrying a load of up to 5 *kg*, achieves a translation range of 220 *μm* and a deflection range of 2.0*mrad*, with stepping resolutions of 20 *nm* and 0.19*μrad*.

Building on the working principles of precision positioning platforms and the demand for high resolution and high speed in positioning and pointing applications, this paper proposes a novel piezoelectric-driven positioning and pointing mechanism, which can achieve high speed and high resolution based on resonant and stick–slip driving principles, respectively, to meet the application requirements of positioning and pointing systems.

## 2 Materials and methods

**Figure 1:**
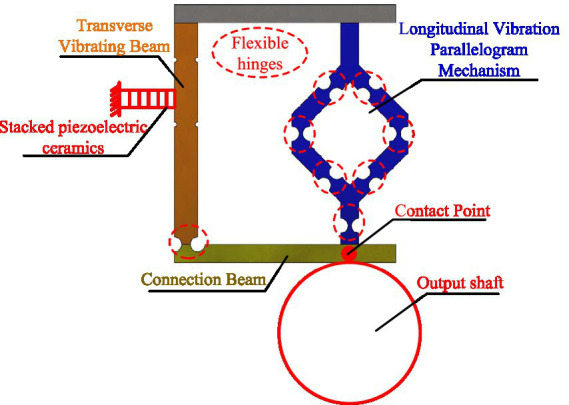
The structure of the drive module.

The drive module of the proposed novel positioning and pointing mechanism in this paper is shown in Figure 1, consisting of a transverse vibrating beam, connection beam, and a longitudinal vibration parallelogram mechanism. The left side of the transverse vibrating beam is rigidly attached to a stacked piezoelectric ceramic, and the output shaft is fixed at the contact point of the connection beam. When a high-frequency excitation is applied to the piezoelectric ceramic, the drive module resonates, with the transverse vibrating beam generating horizontal vibrations and the parallelogram mechanism generating vertical vibrations. The connection beam combines these two vibration directions and generates a two-dimensional elliptical motion at the contact point, thus driving the output shaft to rotate rapidly. In the stick–slip driving mode, the stacked piezoelectric ceramic gradually extends, driving the transverse vibrating beam to displace in the horizontal direction. This displacement is transferred to the contact point via the connection beam, resulting in a slow rotation of the output shaft.

### 2.1 Stick–slip driving principle

**Figure 2:**
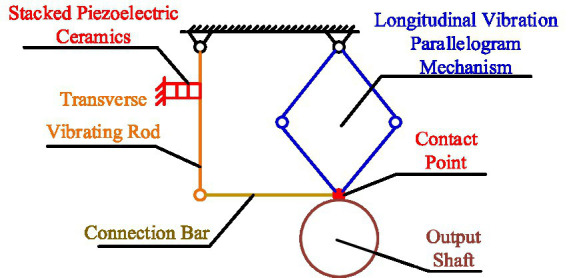
The simplified model of the drive module.

To analyze the motion process of the drive module under stick–slip driving, it is equivalent to the simplified model shown in Figure 2.

**Figure 3:**
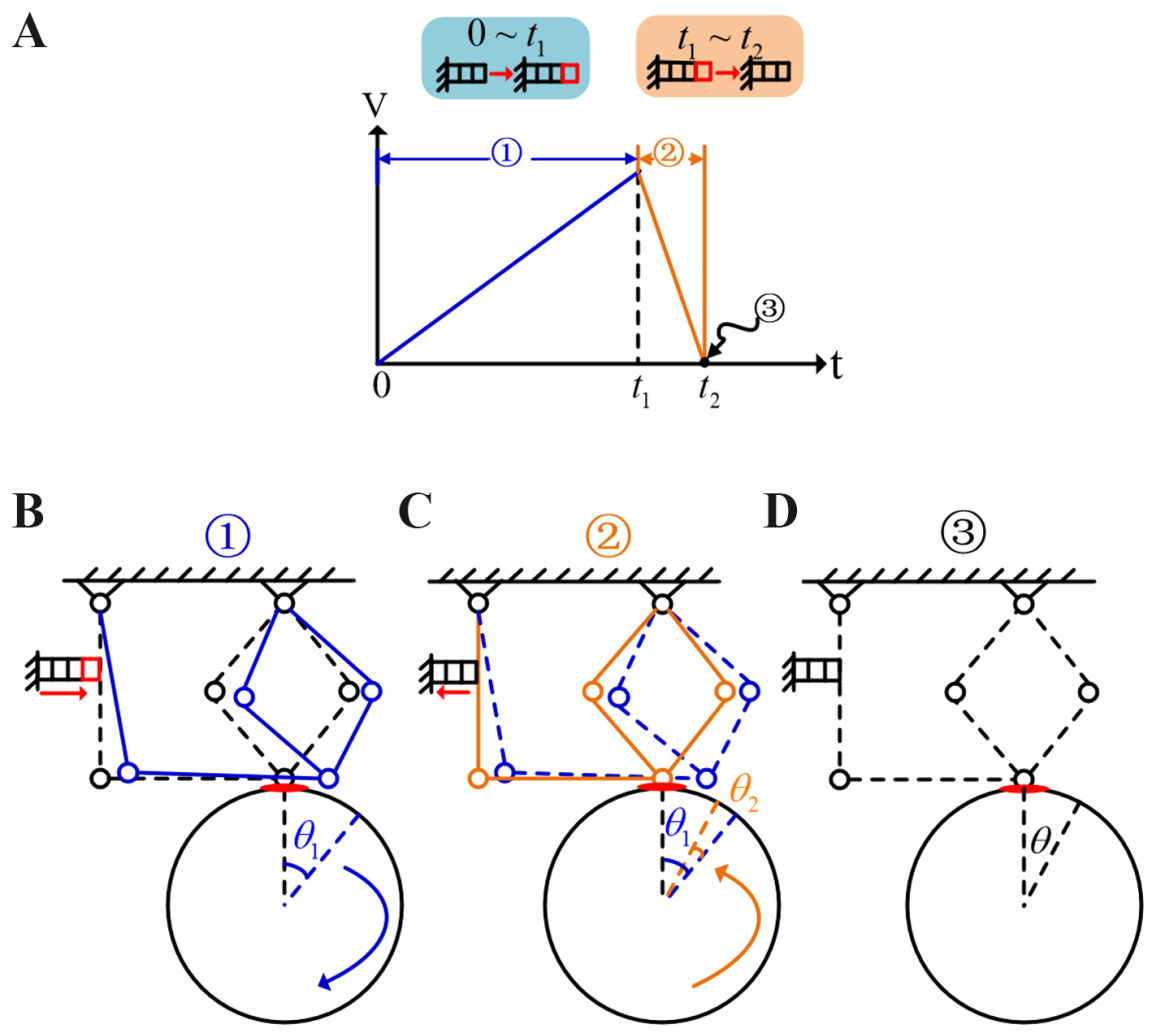
Stick–slip driving principle. **(A)** Low-frequency saw tooth wave. **(B)** The motion state of the drive module at 0 ~ *t*_1._ **(C)** The motion state of the drive module at *t*_1_ ~ *t*_2._ **(D)** The motion state of the drive module at *t* = *t*_2._

When a low-frequency sawtooth wave excitation, as shown in Figure 3A is applied to the piezoelectric ceramics, at t=0 , the excitation voltage is zero, and both the mechanism and the output shaft are in a stationary state. In the $0\sim t_{1}$ stage, the voltage gradually increases, causing the stacked piezoelectric ceramics to slowly elongate. This elongation drives the transverse vibrating beam, causing point A to move to the right. As a result, the connection beam moves point B to the right. Under the influence of friction, the output shaft is driven to rotate by a small angle $\theta_{1}$ , as shown in Figure 3B. At this stage, it can be assumed that the acceleration of the drive module is zero, which means Equations 1, 2:


(1) a=0

(2) ma<mgμ

In the equation, *m* represents the mass of the driving mechanism, and *μ* represents the friction coefficient.

At this moment, the drive module and the output shaft have the same velocity, which is Equation 3:


(3) $$v_{1}=v_{2}=\frac{d_{1}}{t_{1}}$$

In the equation, $v_{1}$ and $v_{2}$ represent the velocities of the drive module and the output shaft, respectively, while $d_{1}$ represents the horizontal displacement of points A or B of the drive module.

The forward rotation angle of the output shaft, denoted as $\theta_{1}$ , is Equation 4:


(4) $$\theta_{1}=\frac{d_{1}}{R}$$

In the equation, *R* represents the radius of the output shaft.

During phase $t_{1}\sim t_{2}$ , when the voltage reaches its maximum value and then quickly drops to zero, the piezoelectric ceramic contracts rapidly. Point A moves quickly to the left, pulling point B to the left as well, as shown in Figure 3C. At this moment, the velocity $v_{1}$ of the drive module is Equation 5:


(5) $$v_{1}=\frac{-d_{1}}{t_{2}}$$

Under the influence of inertia force, the velocity of the output shaft $v_{2}$ remains unchanged, and relative sliding occurs between the drive module and the output shaft. At this moment, the friction force *f* between them is Equations 6, 7:


(6) f=ma

(7) a=gμ

Due to the influence of inertia force, the output shaft generates a reverse rotation angle, denoted as $\theta_{2}$ , which is Equation 8:


(8) $$\theta_{2}=\frac{\int_{0}^{t_{2}}(\int_{0}^{t_{2}}\mathrm{adt}-v_{2})\mathrm{dt}}{R}$$

In summary, at the end of phase $t=t_{2}$ , a complete excitation cycle of the sawtooth wave signal concludes, resulting in a net rotation angle, denoted as $\theta =\theta_{1}-\theta_{2}$ , of the output shaft, as shown in Figure 3D.

Since the output displacement precision of the piezoelectric ceramic can be controlled at the nanoscale, the mechanism can achieve high resolution under low-frequency sawtooth wave excitation.

### 2.2 Simplified dynamics model

**Figure 4:**
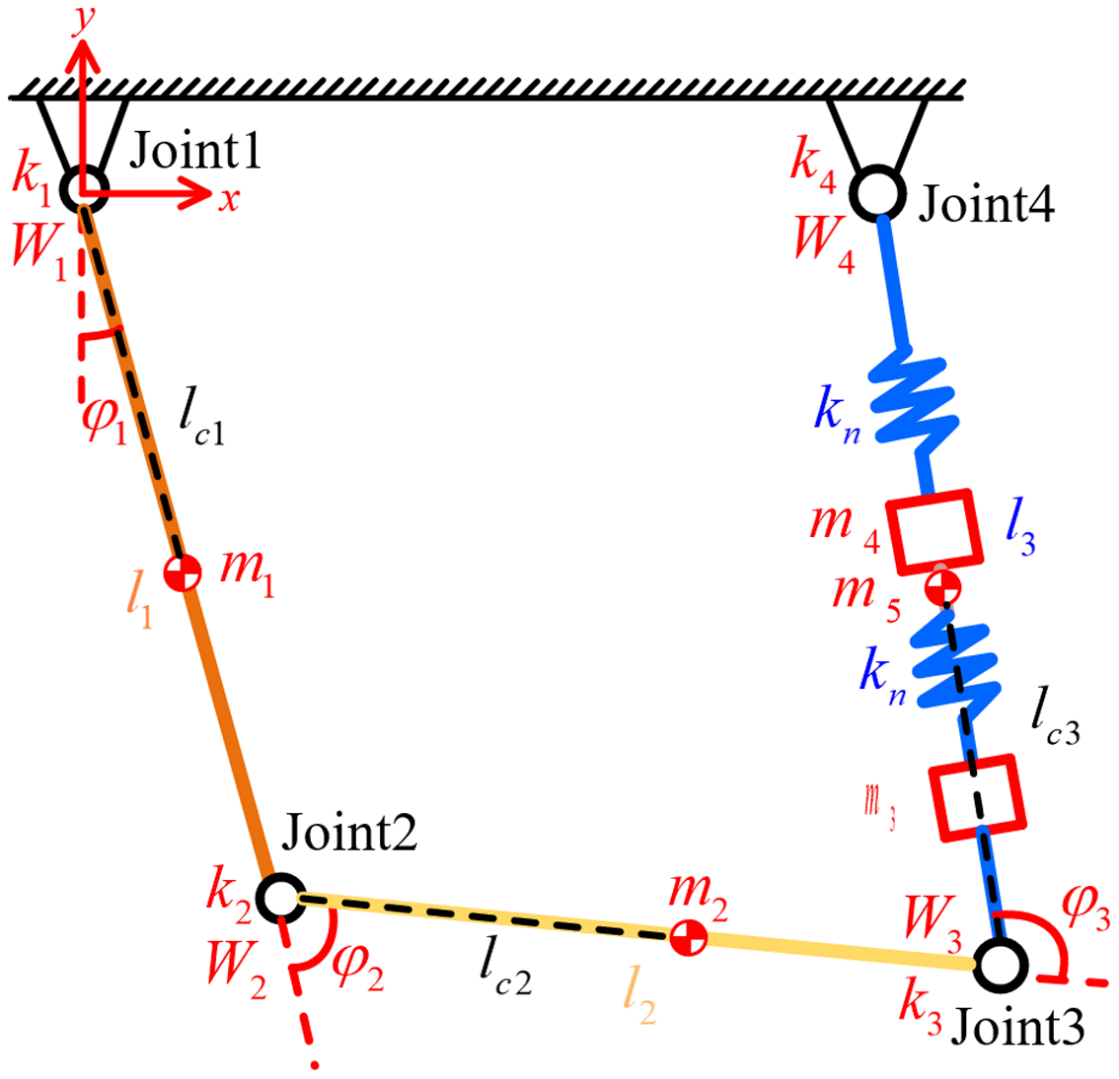
The simplified dynamic model of the drive module.

To specifically analyze the motion characteristics of the drive module under stick–slip driving, its equivalent dynamic model needs to be established. The beam and flexible hinges are replaced by linkages and torsion springs, and the longitudinal vibration parallelogram mechanism is replaced by springs and mass blocks. The resulting simplified dynamic model is shown in Figure 4.

Where: $m_{i}$ (*i* = 1,2,3) represents the mass of the linkages, $m_{i}$ (*i* = 4,5) represents the mass of the mass blocks, $l_{i}$ (*i* = 1,2,3) denotes the length of the linkages, $l_{\mathrm{ci}}$ (*i* = 1,2,3) is the distance from the center of mass of link *i* to the joint *i*, $k_{i}$ represents the joint stiffness, $W_{i}$ is the joint torque, $k_{n}$ is the spring stiffness, and $\varphi_{i}$ is the joint angle.

Due to the presence of impacts within the system, an analysis of the impact forces between the contact point of the drive module and the output shaft is required to assess the influence of impacts on the motion of the mechanism. A nonlinear spring-damping model is used to describe the normal impact forces, as follows is Equation 9


(9) $$\left\{ \begin{array}{l} N=k_{n}{\left(-\delta \right)}^{e}(1+c_{n}(-\dot{\delta })),\delta <0\\ N=0,\delta \geq 0 \end{array}\right.$$

The Coulomb model is used to describe the tangential impact forces, and the arctangent function is employed to model the abrupt change in the impact forces, as follows Equation 10


(10) $$f=\mu \cdot \arctan(\gamma \cdot v_{t})N$$

In the above equations: $k_{n}$ is the normal impact stiffness, $c_{n}$ is the normal impact damping, δ is the impact depth, e is the nonlinearity exponent, μ represents the coefficient of friction, $v_{t}$ is the tangential velocity, and γ is the shape parameter.

The author has previously conducted a force analysis of this simplified model and derived the dynamic equations and parameters for each component. These are not repeated in the present paper.

### 2.3 Stick–slip driving simulation

**Figure 5:**
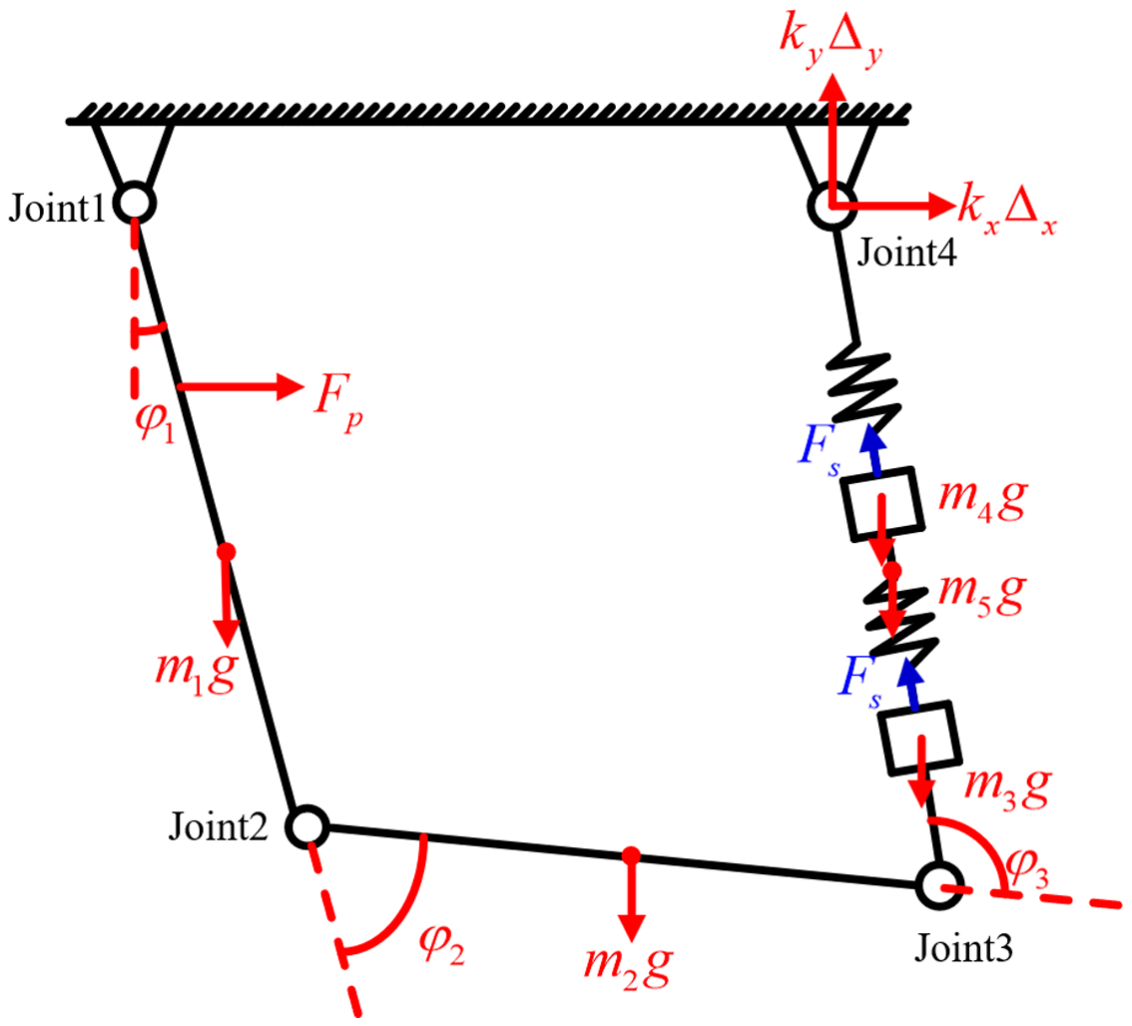
Static analysis of the drive module.

From the above analysis, it can be concluded that when a low-frequency sawtooth wave excitation is applied and the voltage increases slowly, the acceleration of the drive module can be considered zero. In this case, the relationship between the driving voltage and the output shaft rotation angle can be obtained through static analysis, as shown in Figure 5.

Based on the torque equilibrium condition, the expression for the joint angles can be obtained as:


(11) $$\begin{array}{c} m_{1}gl_{c1}\sin\varphi_{1}+m_{2}g(l_{1}\sin\varphi_{1}+l_{c2}\sin\sum_{i=1}^{2}\varphi_{i})\\ +m_{3}{\mathrm{gl}}_{a}+m_{4}{\mathrm{gl}}_{b}+m_{5}{\mathrm{gl}}_{c}-k_{y}\Delta_{y}l_{2}-\frac{l_{1}}{3}F_{p}\cos\varphi_{1}\\ +F_{s}\sin(\sum_{i=1}^{3}\varphi_{i}-\pi )\Delta l_{1}-F_{s}\cos(\sum_{i=1}^{3}\varphi_{i}-\pi )\Delta l_{2}=k_{1}\varphi_{1} \end{array}$$

(12) $$\begin{array}{c} m_{2}gl_{c2}\sin\sum_{i=1}^{2}\varphi_{i}+m_{3}gl_{b1}+m_{4}gl_{b2}+m_{5}gl_{b3}\\ -m_{1}g(l_{1}-l_{c1})\sin\varphi_{1}+\frac{2l_{1}}{3}F_{p}\cos\varphi_{1}\\ -k_{y}\Delta_{y}(l_{2}\sin\sum_{i=1}^{2}\varphi_{i}-{l_{4}}^{'}\sin(\sum_{i=1}^{3}\varphi_{i}-\pi ))\\ +k_{x}\Delta_{x}({l_{4}}^{'}\cos(\sum_{i=1}^{3}\varphi_{i}-\pi )-l_{2}\cos\sum_{i=1}^{2}\varphi_{i})\\ -F_{s}\sin(\sum_{i=1}^{3}\varphi_{i}-\pi )\Delta l_{3}-F_{s}\cos(\sum_{i=1}^{3}\varphi_{i}-\pi )\Delta l_{4}=k_{2}\varphi_{2} \end{array}$$

(13) $$\begin{array}{c} \sin(\sum_{i=1}^{3}\varphi_{i}-\pi )l_{c1}-m_{5}g\frac{l_{1}}{2}+\sin\sum_{i=1}^{2}\varphi_{i}(-m_{2}g(l_{2}-l_{c2})-l_{2}m_{1}g)\\ -m_{1}g((l_{1}-l_{c1})\sin\varphi_{1}+F_{p}(\frac{2l_{1}}{3}\cos\varphi_{1}+l_{2}\cos\sum_{i=1}^{2}\varphi_{i})\\ +k_{x}\Delta_{x}(l_{1}+2\mathrm{\Delta l})\cos(\sum_{i=1}^{3}\varphi_{i}-\pi )-F_{s}\sin^{2}(\sum_{i=1}^{3}\varphi_{i}-\pi )(l_{1}+\mathrm{\Delta l})\\ +F_{s}\cos^{2}(\sum_{i=1}^{3}\varphi_{i}-\pi )(l_{1}+\mathrm{\Delta l})=k_{3}\varphi_{3} \end{array}$$

Where:

$\Delta l_{1}=2l_{1}\cos\varphi_{1}+2l_{2}\cos\sum_{i=1}^{2}\varphi_{i}-(l_{1}+\mathrm{\Delta l})\cos(\sum_{i=1}^{3}\varphi_{i}-\pi )$ ;
$\Delta l_{2}=2l_{1}\sin\varphi_{1}+2l_{2}\sin\sum_{i=1}^{2}\varphi_{i}-(l_{1}+\mathrm{\Delta l})\sin(\sum_{i=1}^{3}\varphi_{i}-\pi )$ ;
$\Delta l_{3}=(l_{1}+\mathrm{\Delta l})\cos(\sum_{i=1}^{3}\varphi_{i}-\pi )-l_{2}\cos\sum_{i=1}^{2}\varphi_{i}$ ;
$\Delta l_{4}=l_{2}\sin\sum_{i=1}^{2}\varphi_{i}-(l_{1}+\mathrm{\Delta l})\cos(\sum_{i=1}^{3}\varphi_{i}-\pi )$ ;
$l_{a1}=l_{1}\sin\varphi_{1}+l_{2}\sin\sum_{i=1}^{2}\varphi_{i}-\frac{l_{1}}{3}\sin(\sum_{i=1}^{3}\varphi_{i}-\pi )$ ;
$l_{a2}=l_{1}\sin\varphi_{1}+l_{2}\sin\sum_{i=1}^{2}\varphi_{i}-\frac{2l_{1}}{3}+\Delta_{l}\sin(\sum_{i=1}^{3}\varphi_{i}-\pi )$ ;
$l_{a3}=l_{1}\sin\varphi_{1}+l_{2}\sin\sum_{i=1}^{2}\varphi_{i}-\frac{l_{1}}{2}+\Delta_{l}\sin(\sum_{i=1}^{3}\varphi_{i}-\pi )$ ;
$l_{b1}=l_{2}\sin\sum_{i=1}^{2}\varphi_{i}+\frac{l_{1}}{3}\sin(\sum_{i=1}^{3}\varphi_{i}-\pi )$ ;
$l_{b2}=l_{2}\sin\sum_{i=1}^{2}\varphi_{i}+{l_{2}}^{'}\sin(\sum_{i=1}^{3}\varphi_{i}-\pi )$ ;
$l_{b3}=l_{2}\sin\sum_{i=1}^{2}\varphi_{i}+{l_{3}}^{'}\sin(\sum_{i=1}^{3}\varphi_{i}-\pi )$ ;

$l_{c1}=-m_{4}g\frac{2l_{1}}{3}-\Delta_{l}-\frac{l_{1}}{3}m_{3}g+\Delta_{l}+k_{y}\Delta_{y}(l_{1}+2\mathrm{\Delta l})$ _._

Moreover, the equivalent thrust $F_{p}$ of the stacked piezoelectric ceramic is given by: $F_{p}=k_{p}\alpha d_{33}U_{p}(t)$ , where $k_{p}$ is the equivalent stiffness of the stacked piezoelectric ceramic, α is the linear expansion coefficient of the piezoelectric element, $d_{33}$ is the piezoelectric constant, and $U_{p}(t)$ is the excitation voltage.

Based on the geometric relationship, the horizontal displacement $L_{x}$ of joint 3 can be expressed as:


(14) $$L_{x}=l_{1}\sin\varphi_{1}+l_{2}\sin(\varphi_{1}+\varphi_{2})-l_{2}$$

The resulting rotation angle of the output shaft, θ_α_, is:


(15) $$\theta_{\alpha }=\frac{L_{x}}{R}$$

**Figure 6:**
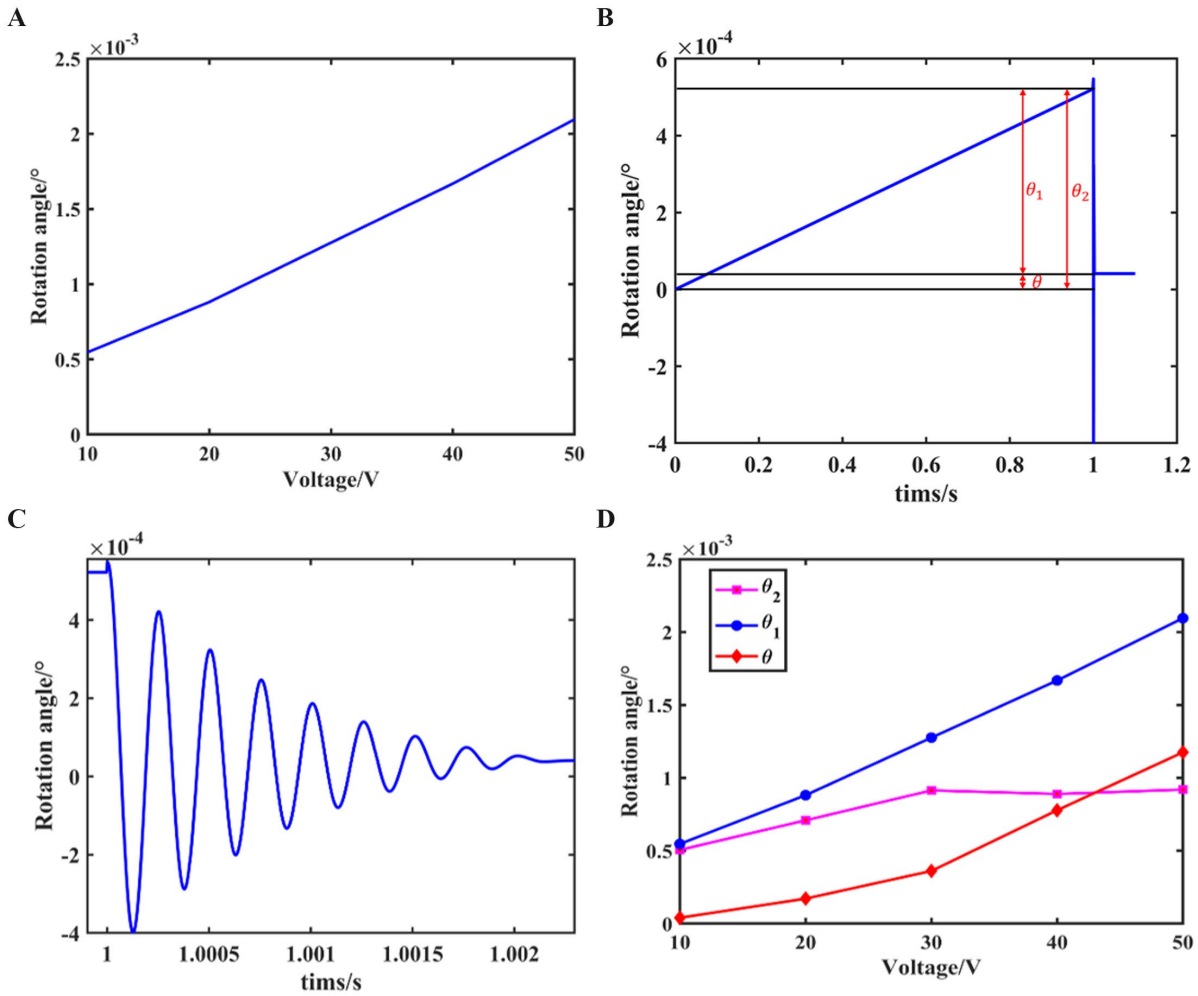
The simulation results of the stick–slip driving principle. **(A)** Relationship between driving voltage and the output shaft rotation angle. **(B)** Variation curve of the output shaft rotation angle within one drive cycle. **(C)** Local enlarged view of panel **(B)**. **(D)** Variation curve of resolution with driving voltage.

In Equations 11–13, $\varphi_{i}(i=1,2,3)$ only depends on $F_{p}$ , and therefore, $L_{x}$ and $\theta_{\alpha }$ are solely functions of the excitation voltage. By solving Equations 11–13, the joint angles $\varphi_{i}$ at different voltage amplitudes can be obtained. These joint angles can then be substituted into Equations 14, 15 to determine the relationship between the excitation voltage amplitude and the output shaft rotation angle $\theta_{\alpha }$ during the phase of slow voltage increase, as shown in Figure 6A.

From the figure, it can be seen that during the slow voltage increase phase of the sawtooth wave excitation, the output shaft rotation angle exhibits an approximately linear relationship with the voltage amplitude.

When the driving voltage increases to its maximum value and then suddenly drops to zero, relative sliding occurs between the drive module and the output shaft. Using the joint angles at the end of the slow voltage increase phase as initial conditions, the dynamic equations are substituted, and coupled with the impact model, the variation trend of the output shaft rotation angle during this phase can be obtained. By combining this with the rotation angle variation curve from the static analysis in the stick phase, a curve representing the angle variation over one excitation cycle is plotted, as shown in Figure 6B, with a local enlarged view provided in Figure 6C.

From the figure, it can be observed that the output shaft rotation angle increases linearly during the slow voltage rise phase. When the voltage abruptly drops to zero, the output shaft rotation angle undergoes a brief oscillation and then experiences a retreat, ultimately resulting in a net rotation angle over one drive cycle.

By selecting driving voltage amplitudes ranging from *10 V ~ 50 V* and repeating the above steps, the relationship between the forward rotation angle $\theta_{1}$ , the retreat angle $\theta_{2}$ generated when the voltage abruptly drops to zero, and the net rotation angle θ with respect to the driving voltage is obtained, as shown in Figure 6D. From the figure, it can be observed that when the driving voltage amplitude is small, the forward rotation angle $\theta_{1}$ is also small and increases linearly with the voltage amplitude. During the process of increasing the driving voltage amplitude, the change in $\theta_{2}$ is relatively small, while the net rotation angle θ increases with the driving voltage.

## 3 Experimental prototypes and experiments

**Figure 7:**
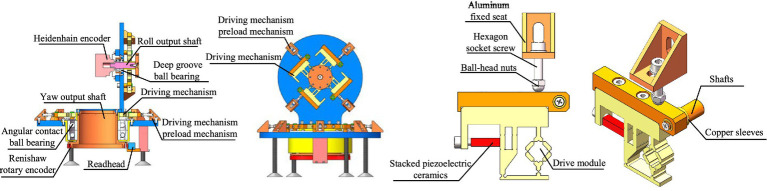
Experimental prototype model.

To validate the analytical results, a dual-axis positioning and pointing mechanism prototype is developed, as shown in Figure 7. The prototype mainly consists of rolling and azimuth output shafts, their respective fixed seats, drive modules, preloading mechanism for the drive modules, deep groove ball bearings, angular contact ball bearings, Heidenhain encoder, Renishaw encoder, and a reading head. The azimuth output shaft is driven by eight drive modules, while the rolling output shaft is driven by four drive modules. The Heidenhain encoder and Renishaw encoder used to measure the rotation angles of the rolling and azimuth output shafts, respectively. The preloading mechanism for the drive modules consists of an angle aluminum fixed seat, screws, and ball-head nuts. The drive modules are fixed to the output shaft seat using copper sleeves and shafts. The preload force between the drive modules and the output shafts can be adjusted by the ball-head nuts.

**Figure 8:**
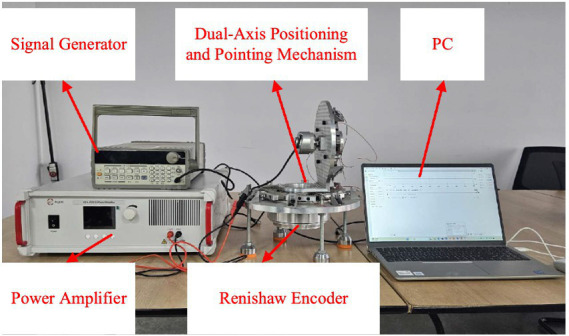
Experimental platform.

The experimental platform is shown in Figure 8 and consists of a signal generator, power amplifier, dual-axis positioning and pointing mechanism prototype, Renishaw encoder, and a PC. The signal generator and power amplifier are used to generate and amplify the sawtooth wave signal, while the Renishaw encoder is employed to detect the rotation angles of the output shafts.

The angle measurement device primarily consists of the Renishaw encoder, the reading head, and the angle acquisition circuit. The encoder is the RESA30USA150B, paired with the RA26BAA150B30A reading head. When using the BISSC protocol, the system achieves a precision of up to 32bits. The chip of the angle acquisition circuit board is the EP4CE6F17C8. The data collected by the reading head is decoded by the angle acquisition circuit board and transmitted to the PC via serial communication for subsequent data analysis. This experiment primarily tests the rotation angle and resolution of the azimuth output shaft driven by the eight drive modules.

### 3.1 Synchronized control experiment

**Figure 9:**
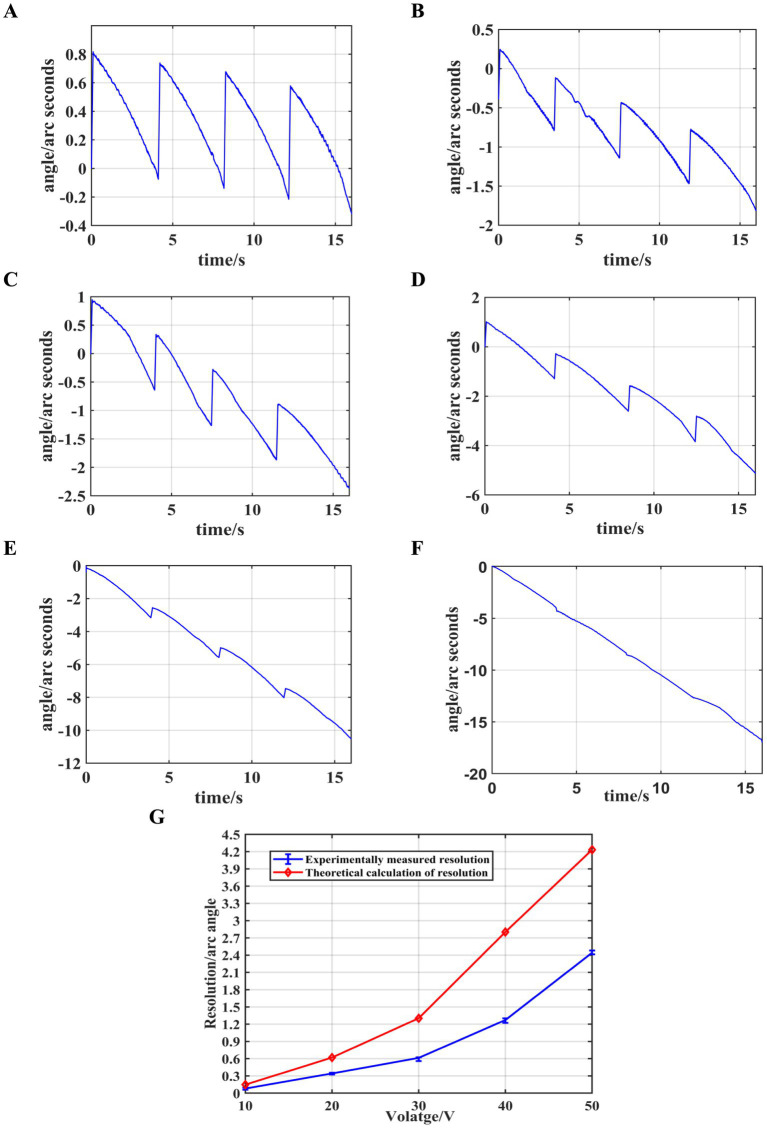
Synchronized control experiment results. **(A)** Angle variations at 10 *V*. **(B)** Angle variations at 20 *V*. **(C)** Angle variations at 30 *V*. **(D)** Angle variations at 40 *V*. **(E)** Angle variations at 50 *V*. **(F)** Angle variations at 60 *V*. **(G)** Resolution at different voltages.

Firstly, a sawtooth wave signal with a period of 4 *s* is generated by the signal generator, amplified by the power amplifier, and then connected in parallel to the piezoelectric ceramics in the eight drive modules, causing them to act synchronously. By adjusting the voltage amplification factor, the excitation voltage amplitude is initially set to 10 *V* and gradually increased in steps of 10 *V* up to 60 *V*. The changes in the output shaft angle and resolution at different voltages are measured, and the results are shown in Figure 9.

As shown in the figure, during the rising phase of the sawtooth wave signal, the stacked piezoelectric ceramics slowly elongate, causing the output shaft to rotate in the forward direction. When the sawtooth wave signal rapidly decreases, a small backward rotation of the output shaft occurs. The net rotation of the output shaft during one excitation cycle is the sum of the forward and backward rotations. It can also be observed that when the driving voltage is 10 *V*, the difference between the forward and backward rotation angles is minimal. As the driving voltage increases, the forward rotation angle increases, while the backward rotation angle remains almost unchanged. At 60 *V*, the backward rotation angle is negligible, and the angle change curve approximates a straight line. When the driving voltage is 10 *V*, the system achieves its highest resolution of 0.079″ ≈ 0.38*μrad*, and the resolution decreases as the driving voltage increases. The trend of resolution variation observed in the theoretical calculations is consistent with the experimental results, confirming the validity of the slip–stick driving theoretical analysis.

However, as observed in this experiment, a significant “backlash” phenomenon occurs during the slip–stick driving tests when using the synchronous control of multiple drive modules, which affects the resolution of the output shaft. Therefore, this study will further improve the stick–slip driving method by employing independent control of multiple drive modules, optimizing their drive phase differences, and examining the changes in resolution.

### 3.2 Independent control experiment

**Figure 10:**
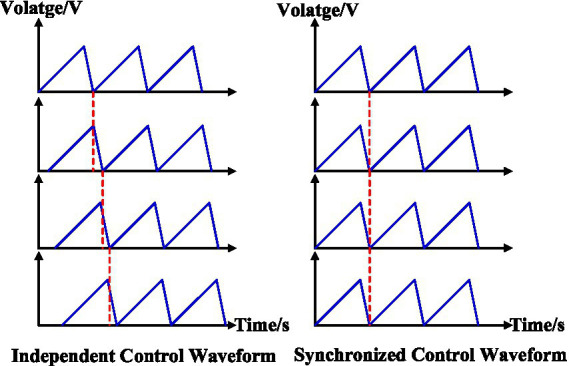
Independent and synchronized control waveform.

To achieve independent control of the drive modules, a sawtooth wave signal with a phase difference of one-eighth is applied to the eight drive modules. This ensures that only one signal undergoes a rapid drop at any given time, causing the corresponding piezoelectric ceramic to retract. The driving waveform is shown in Figure 10.

The signal generation circuit for the independent control experiment consists of a signal generation module, a DAC conversion module, and a filtering and operational amplifier module. The signal generation module utilizes the EP4CE10F17C8N control chip to generate eight sawtooth wave signals based on the DDS (Direct Digital Synthesis) principle. The DAC module employs the AD9708 for digital-to-analog conversion. The analog signals are then passed through a seventh-order Butterworth low-pass filter before being fed into the operational amplifier module. The operational amplifier, selected as the OPA541, performs power amplification on the sawtooth wave signals, which are then output to the piezoelectric ceramics.

**Figure 11:**
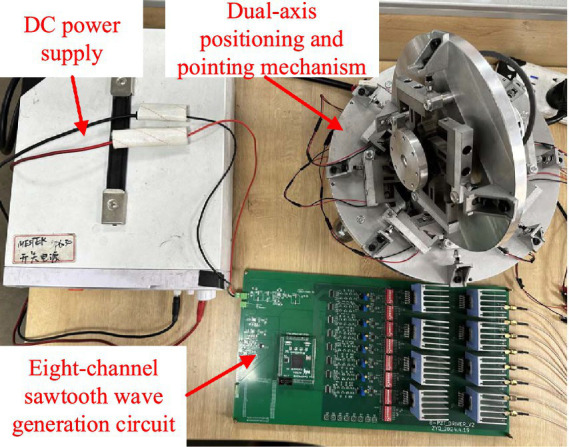
Resolution testing experimental platform.

The waveform generation circuit described above can produce eight sawtooth wave signals with a phase difference of one-eighth. The improved driving waveform is now used to perform resolution tests on the modified dual-axis positioning and pointing mechanism. The experimental platform is shown in Figure 11.

**Figure 12:**
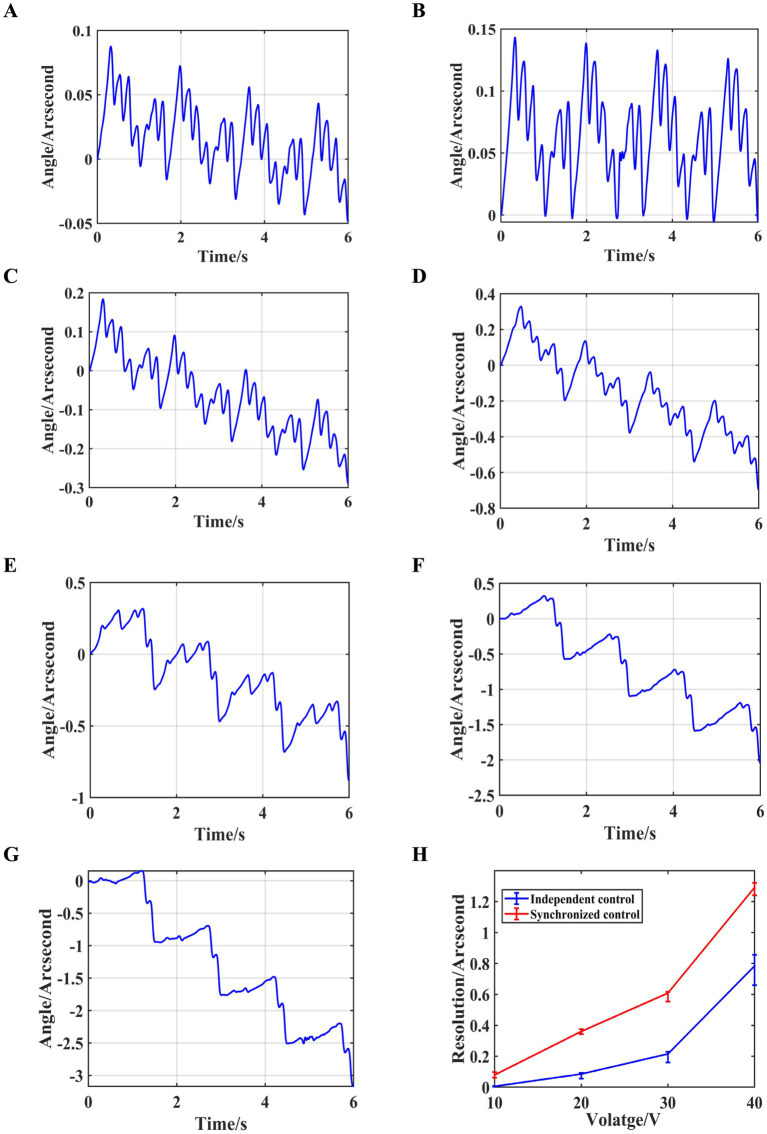
Independent control experiment results with a preload force of 50 *N*. **(A)** Angle variations at 10 *V*. **(B)** Angle variations at 15 *V*. **(C)** Angle variations at 20 *V*. **(D)** Angle variations at 25 *V*. **(E)** Angle variations at 30 *V*. **(F)** Angle variations at 35 *V*. **(G)** Angle variations at 40 *V*. **(H)** Resolution at different voltages.

The period of the eight sawtooth wave excitation signals is set to 2 *s*, with a driving voltage amplitude ranging from 10 *V* ~ 40 *V*, in 5 *V* increments. The preload force between the drive module and the output shaft is adjusted to approximately 50 *N* by rotating the ball-head nut using a torque wrench, and the rotational angle and resolution of the output shaft at different voltages is measured, as shown in Figure 12.

As can be seen from the figure, when independent control is applied with a preload force of approximately 50 *N* and a driving voltage amplitude of 10 *V*, the resolution of the output shaft reaches 0.0057″ ≈ 0.0276*μrad*. Furthermore, the resolution under independent control (0.0057″ ≈ 0.0276*μrad*) shows a significant improvement compared to the resolution under synchronous control 0.079″ ≈ 0.38*μrad*.

**Figure 13:**
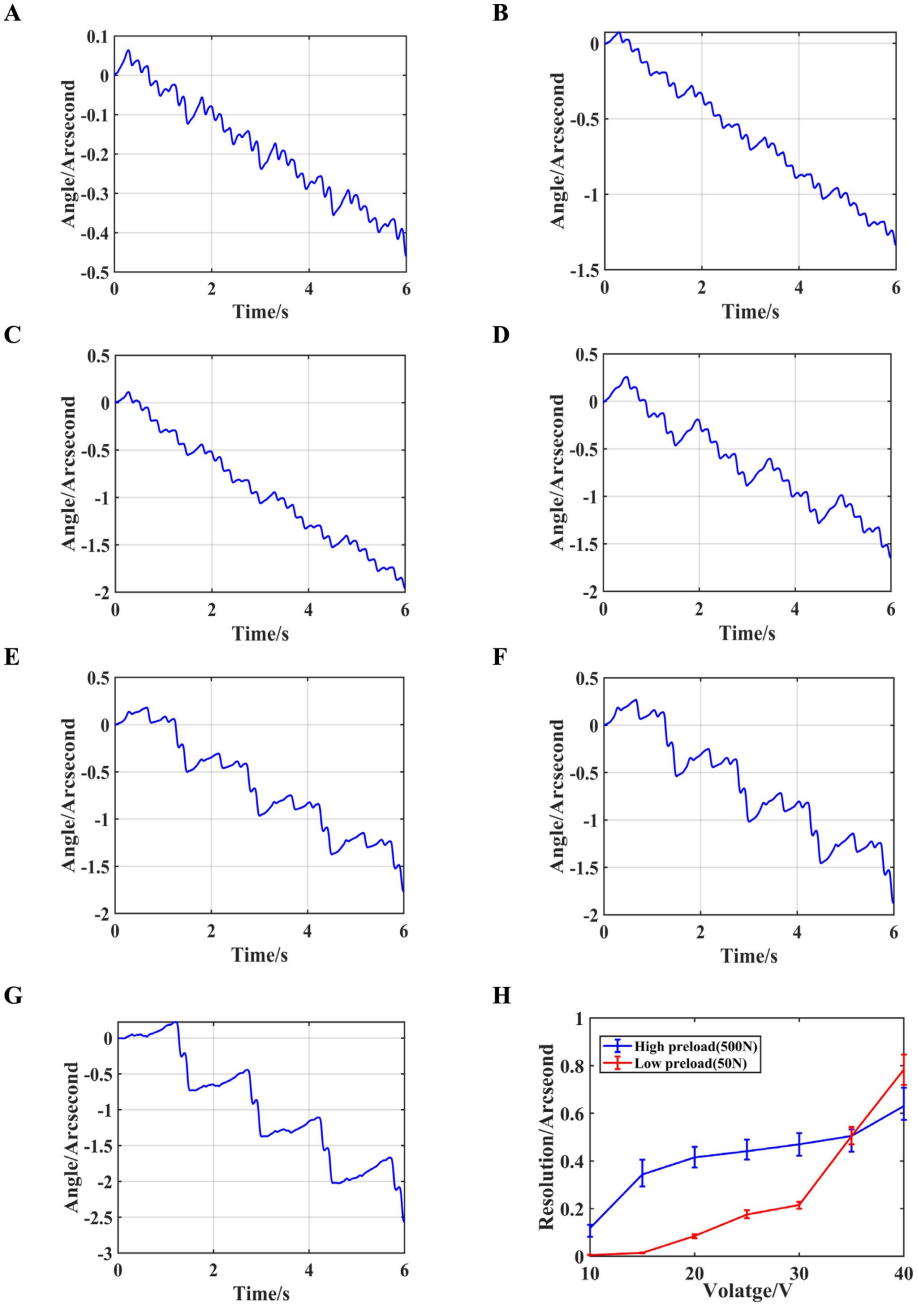
Independent control experiment results with a preload force of 500 *N*. **(A)** Angle variations at 10 *V*. **(B)** Angle variations at 15 *V*. **(C)** Angle variations at 20 *V*. **(D)** Angle variations at 25 *V*. **(E)** Angle variations at 30 *V*. **(F)** Angle variations at 35 *V*. **(G)** Angle variations at 40 *V*. **(H)** Resolution at different voltages.

To investigate the relationship between resolution and preload force, the preload force between the drive module and the output shaft is further increased to approximately 500 *N*. The excitation signal period is still set to 2 *s*, and the voltage amplitude ranged from 10 *V* ~ 40 *V* with a step size of 5 *V*. The rotational angle and resolution of the output shaft and resolution under different driving voltages is measured, as shown in Figure 13.

As shown in the figure, at lower driving voltages, the output shaft’s rotational angle exhibits better linearity with a preload force of 500 *N* compared to 50 *N*. However, as the driving voltage increases, the linearity decreases. Additionally, at low driving voltages, the 50 *N* preload force corresponds to higher resolution. As the voltage increases, the resolution for both preload forces gradually converges.

## 4 Conclusion and discussion

In this work, we introduce a novel positioning and pointing mechanism driven by piezoelectric ceramics, which achieves high resolution based on the stick–slip driving principle. The configuration of the drive module is first introduced, and a simplified dynamic model is established. The motion process within a single sawtooth wave excitation cycle is divided into two phases: the stick phase and the slip phase. Static and transient dynamic analyses are conducted for both phases, revealing the variation trends of the output shaft angle and resolution under different excitation voltage. A prototype of the dual-axis positioning and pointing mechanism, consisting of the drive modules, is developed. Experiments are conducted using both synchronized and independent control of multiple drive modules. When synchronized control is applied with a voltage of 10 *V*, the resolution of the output shaft reaches 0.38*μrad*. When independent control is used with a driving voltage of 10 *V*, the resolution is significantly improved to 0.0276*μrad*. The experimental results confirm that the proposed positioning and pointing mechanism can achieve high resolution based on the stick–slip driving principle. Furthermore, when employing independent control of multiple drive modules, the resolution is notably enhanced compared to synchronized control.

Although the positioning and pointing mechanism proposed in this paper has demonstrated excellent performance in various experiments, several limitations and potential challenges remain that warrant further investigation in future research. First, the classic sawtooth wave is still used as the excitation waveform for the stick–slip driving in this study. Future work could focus on optimizing the drive waveform; by employing an appropriate waveform, the backlash can be further reduced, thereby enhancing the resolution. Second, a phenomenon observed in the experiments is the variation in the output shaft’s rotation angle and resolution with changes in the preload force, which has not been thoroughly investigated. Further research can involve simulation analysis of the output shaft’s resolution and angle variation under different preload forces, potentially identifying the optimal preload range. This could lead to improvements in the linearity of the output shaft’s rotation angle and resolution. In addition, this driving method can be easily applied to the end effectors of robots that require precise operations, and compared to traditional motor-driven methods, its precision will be significantly improved.

## Data Availability

The original contributions presented in the study are included in the article/supplementary material, further inquiries can be directed to the corresponding author.
